# Nevirapine-induced Stevens-Johnson syndrome in children living with HIV in South Africa

**DOI:** 10.4102/sajhivmed.v22i1.1182

**Published:** 2021-02-23

**Authors:** Jacques D. du Toit, Koot Kotze, Helene-Mari van der Westhuizen, Taryn L. Gaunt

**Affiliations:** 1 HIV Outpatient Department, Zithulele Hospital, Mqanduli, South Africa; 2 MRC/Wits Rural Public Health and Health Transitions Research Unit, School of Public Health, University of the Witwatersrand, Johannesburg, South Africa; 3 Nuffield Department of Primary Healthcare Sciences, University of Oxford, Oxford, United Kingdom

**Keywords:** nevirapine, Stevens-Johnson syndrome, toxic epidermal necrolysis, paediatric, HIV, stock-outs

## Abstract

**Background:**

Although adverse drug reactions resulting from the use of nevirapine (NVP) are well described in adults (estimated frequency of 6% – 10%), it has previously been considered less common in children (0.3% – 1.4%). Stock-outs of antiretroviral agents occur frequently in South Africa and result in interruptions in therapy and drug substitutions.

**Objectives:**

To report on a case series of paediatric patients who suffered cutaneous drug reactions to NVP at rates not previously described in children.

**Method:**

We describe a retrospective observational case series of six children living with HIV who developed Stevens-Johnson Syndrome (SJS) following exposure to NVP because of a prolonged stock-out of efavirenz 200 mg tablets in South Africa.

**Results:**

Of the 392 paediatric patients receiving antiretroviral therapy at the institution, 172 were affected by the efavirenz stock-out. Of these, 85 children were changed to NVP of which six developed NVP-induced SJS (7.1% incidence rate). The median time between initiating NVP and developing symptoms was 27 days (range 12–35 days). All patients responded well to NVP cessation and symptomatic treatment. One patient was referred for specialist care. Two patients were successfully rechallenged with efavirenz after developing SJS and three continued lopinavir/ritonavir.

**Conclusions:**

This is the second largest case series of NVP-induced SJS in children to date and raises the possibility that the incidence of SJS in children may be higher than previously described. Further research is required to explore the risk factors associated with NVP-induced SJS in children. This case series highlights the negative impact of drug stock-outs on patient health outcomes.

## Introduction

Stevens-Johnson Syndrome (SJS) and its severe form, toxic epidermal necrolysis (TEN), are mucocutaneous reactions associated with significant morbidity and mortality.^1^ Stevens-Johnson syndrome/toxic epidermal necrolysis may be caused by a wide range of medications and infections, and in many cases, it may be difficult to definitively establish the causative agent.^2^

Stevens-Johnson syndrome and TEN are thought to be delayed hypersensitivity reactions resulting from interactions between the drug and the adaptive immune system via specific human leukocyte antigen (HLA) subtypes and T-cell receptors.^3^ These drug metabolites likely result in immune mediated apoptosis of keratinocytes and eventual epidermal detachment.^4^ The time between exposure to a causative agent and onset of symptoms is estimated to be between 4 and 28 days.^2^ It has also been hypothesised that human immunodeficiency virus (HIV) infection itself may increase the risk of developing SJS/TEN.^1,5^

The non-nucleoside reverse transcriptase inhibitor (NNRTI) nevirapine (NVP) is a well-established cause of SJS/TEN in adults but is thought to be uncommon in children.^3^ We accessed nine reports of SJS/TEN in children exposed to NVP describing 29 patients in total.^6,7,8,9,10,11,12,13,14^ Although NVP hypersensitivity has been associated with higher CD4 cell counts in adults (> 250 cells/µL in females and > 400 cells/µL in males), the above case reports show SJS and TEN occurring at any stage of immune suppression in paediatric populations.^8^ It has been suggested that the paucity of cases reporting on the association between NVP in children and SJS/TEN may be a result of prescribing behaviour rather than an actual decreased risk compared with adults.^2^ However, there is widespread use of NVP in infants as part of prevention of mother-to-child transmission (PMTCT) programmes and we could only find reference to two cases of children below 1 year developing SJS following exposure to NVP.^8^ The reasons for this remain unclear, although greater immune tolerance in neonates may play a role.

It is estimated that 6% – 10% of adults exposed to NVP will develop some form of drug-induced hypersensitivity, which may be grouped into one of five phenotypes: NVP-induced rash, hypersensitivity syndrome, SJS, toxic epidermal necrolysis, and drug-induced liver injury.^15^ The estimated risk for SJS/TEN in drug safety profile studies in adult and paediatric patients with HIV on NVP-containing regimens was previously thought to be approximately 0.3%.^16^ This contrasts with the estimated 1.4% incidence encountered by the largest case series of NVP associated SJS/TEN in pediatric patients to date.^8^

The National Consolidated Guidelines for PMTCT and the management of HIV in children, adolescents and adults, published by the Department of Health of South Africa in 2015 lists efavirenz (EFV) as the preferred NNRTI in children (> 4 weeks old) and adults, but allows for the use of NVP in first line regimens in neonates and in adolescents where EFV is contraindicated. It does not specify CD4 cell counts or percentages at which the use of NVP in antiretroviral (ARV) regimens of children is contraindicated.^17^ The updated 2019 guideline recommends switching children to Dolutegravir-based regimens in South Africa and does not provide recommendations on the use of NVP.^18^ A NVP-containing regimen is still recommended when initiating ART during the neonatal period and further information about potential risks and benefits for its use in children are important, as drug stock-outs lead to clinicians making individualised treatment decisions based on available ARVs.

Stock-outs of antiretroviral treatment, defined by the World Health Organization as the complete unavailability of a specific drug at a delivery or storage point for at least 1 day, are common in South Africa’s antiretroviral therapy (ART) programme and may lead to worse health outcomes and virological failure.^19,20^ Stock-outs disrupt HIV care programmes and pose challenging treatment decisions when the preferred drug is not available. After an adverse event, it is also difficult to decide whether a drug in the same class can be re-introduced; although it has been shown that it is safe to restart EFV in patients who had SJS because of NVP.^21^ In this article we describe SJS that developed in children after switching to NVP during an EFV stockout.

## Methods

### Background

During February to May 2018 there was a prolonged stock-out of 200 mg EFV tablets at a rural hospital in the Eastern Cape, South Africa. A strategy was developed by the Acting Clinical Manager and Head Pharmacist in conjunction with a paediatric infectious disease specialist to limit the effects on patient care. Children were switched to the best possible alternative treatment regimens, taking drug stock levels and individual case details into account. A viral load cut-off was used to split the large cohort of children into those who were fully or partially suppressed and those who had a higher level of viraemia. A viral load of 400 copies/mL was used as this was the lowest level of viral suppression that the laboratory could accurately measure in the period preceding the stock-out. There were also limited supplies of EFV 50 mg tablets and lopinavir/ritonavir (LPV/r), so it was not possible to transition the entire cohort to these alternatives. Children with a viral load deemed acceptable given the circumstances (< 400 copies/mL in the preceding 6 months) were switched to either NVP or dispensed the required EFV dose using 50 mg tablets. As a result of the the concern of rapidly depleting the EFV 50 mg tablet supply and further disrupting treatment in younger children, older children were preferentially changed to NVP. If children were not adequately virally suppressed (> 400 copies/mL in the preceding 6 months), they were changed to second line therapy and thus received LPV/r. Children who were on 400 mg EFV were switched to 400 mg immediate release (IR) NVP nocte and those on 300 mg EFV were switched to IR NVP 200 mg in the morning and 100 mg in the evening based on local institutional practice.

### Study design

This study is a retrospective observational case series describing the clinical course of a cohort of children living with HIV who developed an adverse drug reaction after substitution of their antiretroviral drugs. The researchers had no prior intention to evaluate the effects of NVP in this population preceding the substitution and the decision to write up the cases was made retrospectively. Clinical and drug prescription information was extracted from routinely collected HIV service programmatic data and paediatric ward admission notes. Contextual information regarding the HIV programme and treatment history was obtained through the iDART (intelligent Dispensing of ART) electronic record keeping system.

There are no universally accepted diagnostic criteria for SJS/TEN. A minimum of two of the following clinical features with the history of new exposure to NVP were used as inclusion criteria for this case series: prodrome of acute-onset febrile illness and malaise; painful rash that progresses rapidly; erythematous macules, targetoid lesions, vesicles, or bullae; positive Nikolsky sign; and mucosal involvement.^22^ Total Body Surface Area (TBSA) percentage involvement was documented using the Lund and Browder Chart to distinguish between SJS (< 10%), SJS/TEN overlap (10% – 30%), and TEN (> 30%)^23^.

### Ethical consideration

Ethical approval was granted from the Human Research Committee at Walter Sisulu University’s Faculty of Health Sciences Postgraduate Education, Training, Research and Ethics Unit (Ref: 022/2019). Permission for the study was attained from the relevant institutions and the Eastern Cape Health Research Committee (Ref: EC_201907_013). Written consent was obtained from the primary caregivers of children included in this case series.

## Results

The EFV 200 mg tablet stock-out affected 172 children. This represented 44% of the total paediatric population (age < 15 years) collecting ARV treatment in the hospital catchment area at that time (*n* = 392). Eighty-five patients were switched from EFV to NVP, with the remaining patients either receiving EFV in 50 mg tablets (34 patients), LPV/r (45 patients), sourcing EFV 200 mg tablets elsewhere (3 patients), or were lost to follow-up (5 patients).

Six cases of SJS/TEN (incidence rate = 7.1%, 95% CI: 1.6% – 12.5%) were reported. Five of the six patient’s caregivers consented to link clinical records with this case series, and this review will discuss details of those children. Based on these notes, no other new medication had been prescribed surrounding the introduction of NVP and SJS/TEN was the most likely clinical diagnosis. The mean age of the participants was 10.4 years (SD: 2.9) and the mean weight was 26.8 kg (SD: 4.9) (Table 1). Five of the children were receiving abacavir (ABC)/lamivudine (3TC)/EFV before the drug switch. One child had confirmed prior exposure to NVP through the PMTCT programme. All children were reportedly healthy preceding their exposure to NVP, with no concurrent treatment initiation recorded.

**TABLE 1:** Case information of patients with Stevens-Johnson syndrome/toxic epidermal necrolysis.

Characteristics	Case 1	Case 2	Case 3	Case 4	Case 5	Case 6
Age (years)	7	14	11	12	8	§
Sex	Female	Male	Male	Male	Male	§
Weight (kg)	19	29	30	31	25	§
Original ART initiation	2017/01	2006/09	2009/05	2009/04	2017/02	§
CD4 count at ART initiation	565 (2017/01)	Not available‡	Not available‡	1652 (28%) 2009/04	362 (2016/01)	§
Most recent CD4 count in cells/μL (%, date)	654 (33.3%, 2018/01)	561 (2016/08)	1218 (44.5%, 2015/09)	Not available‡	Not available‡	§
Previous ART regimen	ABC/3TC/EFV	ABC/3TC/EFV	D4T/3TC/EFV then ABC/3TC/EFV	D4T/3TC/EFV then ABC/3TC/EFV	PMTCT NVP then ABC/3TC/EFV	§
Change during stock out	ABC/3TC/NVP	ABC/3TC/NVP	ABC/3TC/NVP	D4T/3TC/NVP	ABC/3TC/NVP	§
NVP dose	200 mg am/100 mg pm	400 mg nocte	400 mg nocte	400 mg nocte	400 mg nocte	§
Days before onset of symptoms	27	12	35	21	28	§
Rash type and distribution	No rash	Generalised rash	Generalised rash	Generalised rash	Generalised rash	§
Classification†	SJS	SJS	TEN	SJS	SJS	§
Mucous membrane involvement	Lip ulceration	Lip ulceration; Conjunctival hyperaemia	Lip and oral ulceration; Ulcerated glans	Conjunctival hyperaemia	Lip ulceration	§
Blood investigations	Normal	ALT 1·5 × Normal	ALT 1·5 × Normal	Normal	Normal	§

ALT, alanine transferase; ART, antiretroviral therapy; D4T, stavudine; ABC, abacavir; 3TC, lamivudine; NVP, nevirapine; EFV, efavirenz; PMTCT, prevention of mother to child transmission; SJS, Stevens-Johnson syndrome; TEN, toxic epidermal necrolysis.

† , Total body surface area: SJS (< 10%); SJS/TEN overlap (10% – 30%); and TEN (> 30%).

‡ , Results not available at point of data extraction.

§ , SJS recorded as adverse outcome on electronic database, but not linked with case notes as caregiver declined participation in case series.

The median time between initiating NVP and developing symptoms was 27 days (range 12–35 days). All patients responded well to NVP cessation, symptomatic treatment, and eye care if applicable. One patient was referred for tertiary care. Two patients were successfully rechallenged with EFV after developing SJS/TEN and the other three continued LPV/r.

## Discussion

Prior to this case series, few cases of SJS/TEN in children after exposure to NVP have been described in the literature, with some experts believing that NVP associated SJS/TEN is uncommon in children living with HIV.^6,7,8,9,10,11,12,13,14^ Our case series showed an incidence rate of 7.1% of SJS/TEN after exposure to NVP because of a stock-out of EFV 200 mg tablets. This was higher than previously estimated (0.3% and 1.4%), however, given the small number of cases, the 95% confidence interval is wide (1.6% – 12.5%), and the true incidence in a larger population may be lower than what we found and closer to these estimates. Our result, and the true estimate, may however be higher than previously estimated and instead be congruent with the estimated incidence of NVP hypersensitivity in adults.^8,16^

We postulate that the reasons for the higher than expected incidence rate may be related to a genetic predisposition to developing NVP-induced SJS/TEN in this cohort of patients. Increasing bodies of literature are describing strong associations between HLA alleles and severe cutaneous adverse reactions with certain drugs.^24^ Although there are limited studies on SJS/TEN in sub-Saharan Africa, there is some evidence linking specific HLA subtypes (such as HLA-Cw*04) to NVP-induced SJS in populations from these regions.^15,25^ Adverse events relating to NVP have also been shown to strongly associate with single nucleotide polymorphisms in genes known to influence the rate of plasma clearance of NVP.^25^

We were unable to perform an analysis to investigate the influence of CD4 cell count on the incidence of SJS in our dataset, and further research could help clarify whether this is a contributing factor in children, as it is a known risk factor for adverse reactions to NVP in adults.^26^

The NVP tablets used were an IR formulation as the 400 mg extended release formulation was not available. Whilst some participants received this formulation as two divided doses, others received this as a single dose. Based on this and SJS being an immunological, dose independent reaction,^27^ we are uncertain of the contribution of this to a higher than expected incidence of SJS in this cohort.

The demographic characteristics of the patients in this case series are similar to those described in the literature. The time to onset of symptoms was similar to that seen in previously described cases of SJS/TEN (4–28 days), with a median of 27 days (range 12–35 days) amongst the patients treated in this series. The most severe case of TEN in our series had the longest interval between switching to NVP and presenting with symptoms (35 days). This could be because of delays in accessing healthcare in a rural setting, rather than an actual extended time before onset of symptoms.

This case series also demonstrates the difficulty of safely managing drug stock-outs, especially in rural healthcare settings. Given the difficulty of switching a large group of patients to a single alternative and the potential for triggering further stock-outs in other medications in a ‘knock-on’ effect, individualised substitutions had to be made in consultation with paediatric HIV experts. Unfortunately, and possibly because of a combination of the given contributing factors, the incidence of NVP associated SJS/TEN in our setting was far greater than previously reported in the literature. Knowledge of this case series may influence risk-benefit assessments in future similar scenarios and assist clinicians in selecting safer alternative regimens for children living with HIV. Although we as authors advise against the use of NVP in this type of patient, stock-outs are an unfortunate reality and NVP may be the only available alternative. If a clinician has exhausted all other options as outlined in their national guidelines (such as the use of Dolutegravir or LPV/r) and a drug holiday is not appropriate, we advise the following approach:

- The patient and their responsible guardian be counselled regarding the potential risks and early warning signs of SJS.
- Increase monitoring frequency (approximately a 2- and 4-week follow-up).
- Avoid addition of other new medication.

## Limitations

Information for this case series was collected retrospectively and not all desired data were available. Furthermore, most of the patients were managed by different clinicians, resulting in differences in diagnostic criteria used and drug histories. The authors made every effort to ensure that the data collected was accurate despite these difficulties.

As a result of the mobility of patients using health services in the region of the study, it is also possible that there were more cases of SJS/TEN or other adverse reactions in the NVP-substitution cohort. These patients may have presented for healthcare elsewhere, although only one patient was lost to follow-up in the NVP-substitution cohort.

## Conclusions

The risk of NVP-induced SJS/TEN in children living with HIV may be significantly higher than previously documented. Medication shortages have a negative impact on HIV care, and strategies to prevent stock-outs should be a key part of health system strengthening. National directives should be made available to assist healthcare workers in safely caring for patients when shortages occur.
